# Milk Sickness

**Published:** 1876-06

**Authors:** 


### MILK SICKNESS.

W. J. Brewington, M.D., of New Corydon, Ind., thus writes of this disease:

There is, perhaps, no disease that prevails to so limited an extent, that has been the subject of so much discussion as the one under consideration; and the ideas as to its causes and nature are as various, almost, as the writers on the subjeet.

Having had an extensive experience in the treatment of this disease for the last ten years, I consider it my duty to give the profession the benefit of some of my observations.

Physicians are pretty much agreed as to symptoms; I shall not, therefore, occupy much space in recording the symptoms that point to a diagnosis; but will consider such only as are pathognomonic o the disease.

1.—A peculiar odor is exhaled from the surface; resembling,

(as Prof. Wood describes it), a mixed smell of chloroform and mercurial salivation.

2.—Violent throbbing of the abdominal aorta throughout its whole extent.

3.—Vomiting of a peculiar character; at first the substances are ejected which were last swallowed; then a dark green fluid, which becomes darker and darker, and finally resembles the coffee grounds vomit of yellow fever.

These symptoms, in a well developed case, are never absent, and with the obstinate constipation, constant restlessness and sighing respiration, cannot fail to furnish a correct diagnosis.

*Causes.*—This question has puzzled the minds of the profession ever since the disease was known to exist; some contending that it results from animals eating a shrub called rhus toxicodendron; others ascribe it to the white snake root; others to malaria, etc.

The first of these opinions I can never endorse, and for various reasons. In first place, there is scarcely a square mile of territory in all this section of country where the rhus does not grow in abundance, and animals feed upon it whenever they can obtain it, but the disease dose not prevail to so great an extent, it being confined to a narrow strip not exceeding two miles in breadth, extending from this place through Adams and Jay counties in Indiana and through Mercer and Auglaize counties in Ohio.

The village of New Corydon is situated on the north bank of the Wabash river, two miles from the Ohio line, where this disease used to prevail to an alarming extent; while on the South side of the little stream a case was never known to occur. Another reason: We meet with this disease almost invariably during a dry season, no matter what season of the year, winter and summer alike. The worst scourge I ever knew in this country was in winter, when there was no foliage for animals to feed upon. This is proof positive that it is not the result of eating poison oak, nor any other vegetable.

The rhus produces effects similar to those of strychnia; hence, it is sometimes employed in the treatment of paralysis. Now, in milk sickness, there is relaxation of the whole system, not a symptom of poisoning by that agent; furthermore, a patient with this disease never dies with convulsions.

As to its *malarial* origin: That idea, I think, should be altogether abandoned. When we consider that all this part of the country is a malarial region, and that the disease called milk sickness is confined within such narrow limits, we need say no more to disprove such a hypothesis.

My opinion is that it is an irritant poison contained in water which produces the disease, both in man and the lower animals. I do not believe that in this vicinity there is one in fifty persons, who take the disease, that gets it by using milk or butter from diseased animals.

In places where the disease used to be most prevalent, the people used well water for cooking and drinking, but since they built cisterns, and used water from them exclusively, they are entirely exempt from the disease.

In a neighborhood, three miles east of this place, patients used to die with milk sickness frequently. It seemed to be attended by a singular fatality in that particular vicinity. Since the use of well water has been abandoned there has not a single case been known. The same is true, so far as I know, of every other place where the same plan has been adopted. I do not pretend to say that the disease is never produced in man from milk or butter, but, so far as my experience goes, those cases are rare exceptions.

*Prognosis.*—This, I consider under the appropriate treatment, to be favorable. During the last seven years I have not lost a single uncomplicated case, but each succeeding attack renders the prognosis more grave.

*Treatment.*—I use but few remedies. The indications are: 1st. To allay vomiting, so that the stomach will retain medicines. 2d. To neutralize the poison in the system. 3d. To arouse the secretions, particularly of the bowels and liver.

To fulfil the first two indications I have found nothing answer so well well as rye whisky; and, in the advanced stages of the disease, there is not much danger of giving too much. I have frequently given half a pint of strong whisky in an hour to a patient unaccustomed to its use. As a rule, an ounce every half hour, until vomiting has ceasesd, is sufficient. But there is no danger of producing the peculiar effects of alcohol until the disease is nearly subdued. When the stomach is sufficiently quiet, the next thing is to clear the bowels, for which purpose I give hyd. sub.-mur. grs. x., and in three hours after follow with an enema, and repeat until the bowels are freely moved. I greatly prefer using injections instead of overloading the stomache with cathartic medicines, as it always requires an intolerable amount of such medicines to act.

This is the plan of treatment I have adopted with uniform success. Of course, complications must be met with appropriate remedies.—*Clinic.*

